# Epidemiological Analysis of Respiratory Diseases in Mexico From 2000 to 2020: Trends, Geographic Distribution, and Public Health Challenges

**DOI:** 10.7759/cureus.76521

**Published:** 2024-12-28

**Authors:** Luz Isela Peinado-Guevara, Mayra Mejía-Sánchez, Ricardo Clark-Tapia, Cecilia Alfonso-Corrado, Samuel Campista-León

**Affiliations:** 1 Faculty of Biology, Autonomous University of Sinaloa, Culiacan, MEX; 2 Institute for Environmental Studies, Sierra Juarez University, Ixtlan de Juarez, MEX

**Keywords:** acute respiratory infections, bronchopneumonia, pandemic, respiratory diseases, seasonal variability

## Abstract

Introduction: In Mexico, respiratory diseases such as tuberculosis (TB), acute respiratory infections (ARI), pertussis (Pt), and pneumonia-bronchopneumonia (Nemu) represent critical public health challenges that contribute to morbidity and mortality and are exacerbated by socioeconomic factors and the COVID-19 pandemic.

Objective: To evaluate the trends, seasonal patterns, and geographic distribution of major respiratory diseases in Mexico between 2000 and 2020.

Methodology: Data from the National Epidemiologic Surveillance System were analyzed using advanced statistical methods, including Kruskal-Wallis tests, Mann-Whitney analysis, and multivariate analysis, to identify temporal and regional variations.

Results: A 21-year analysis revealed significant (χ² = 63.57, p < 0.01) trends in the incidence of respiratory diseases in Mexico. Results showed an increase in TB and Pt cases in recent years and a decreasing trend in ARI and Nemu, with fluctuations influenced by socioeconomic factors and the pandemic. Seasonally, TB and Pt showed the highest incidence in spring and summer (27.4% and 32%, respectively), whereas ARI (29.7%) and Nemu (33%) peaked in winter.

Conclusions: Prevention, early diagnosis and treatment of TB and Pt need to be strengthened, and surveillance of ARI and Nemu must be maintained. Public health policies, vaccination campaigns, and inter-institutional and community collaboration are essential to address these challenges.

## Introduction

Respiratory infections (RI) represent a significant source of global morbidity and mortality, impacting individuals of various ages, with special incidence in children and the elderly [1]. The diversity of causative agents, some well characterized and others yet to be identified, complicates their management and prevention [2,3]. In this context, a detailed understanding of the epidemiological dynamics of RI stands as a fundamental pillar in the face of contemporary health challenges [4].

Numerous studies have addressed the analysis of RI, unraveling both its health and socioeconomic impact [5,6]. For example, a global study by Troeger et al. [7], analyzing data from 195 countries over 25 years, evidenced a worrying vulnerability in children under five years of age. This finding underscores the importance of focusing prevention and treatment efforts on this age group. Likewise, regional research such as that of Sejas Claros and Condori Bustillos [8] in Bolivia, and studies on the relationship between RI and socioeconomic conditions have provided valuable information on risk factors and seasonal patterns of these diseases.

In Mexico, lower respiratory infections have been identified as the predominant cause of morbidity and mortality between 1990 and 2014, with studies in regions such as Chiapas highlighting the impact of inequality in resources and socioeconomic conditions on child health [9,10]. Pneumonia, particularly prevalent in children under five and over 65, emerges as one of the RIs of greatest concern [11].

The national picture also reflects an alarming number of deaths from respiratory diseases, including pneumonia-influenza, chronic obstructive pulmonary disease (COPD), and other pathologies [12]. This study aims to analyze the prevalence of respiratory diseases in Mexico, by federal entity and year, in the period between 2000 and 2020. With this, we aspire to contribute to the design of more effective health policies by identifying areas of vulnerability and temporal trends, thus integrating us into global efforts to mitigate the impact of RI in the general population.

## Materials and methods

Study design and data collection

This retrospective study was based on data mining from epidemiological bulletins available between 2000 and 2020, specifically focusing on four respiratory diseases (RD) classified under the International Classification of Diseases (ICD) 10th revision: pulmonary tuberculosis (TB) (A15), acute respiratory infections (ARI) (J00-J06, J20, J21, excluding J02.2 and J03.0), pneumonia-bronchopneumonia (Nemu) (J12-J18, excluding J18.2, J13, and J14), and pertussis (Pt) (A37). The data were extracted from the National Epidemiological Surveillance System, provided by the General Directorate of Epidemiology [13], offering a comprehensive overview of the status of these RDs at the national level.

Data organization and analysis

Historical information provided by the General Directorate of Epidemiology was consolidated into a database structured by federative entity, and segmented both monthly and annually. The annual and monthly incidence rates per 100,000 inhabitants were calculated for each RD, adjusted by demographic indicators provided by the National Population Commission [14].

Statistical strategies

Mean values and standard deviations were calculated for each month, year, and federative entity [Aguascalientes (AGU, 1), Baja California (BCN, 2), Baja California Sur (BCS, 3), Campeche (CAM, 4), Coahuila de Zaragoza (COA, 5), Colima (COL, 6), Chiapas (CHP, 7), Chihuahua (CHH, 8), Mexico City (CMX, 9), Durango (DUR, 10), Guanajuato (GUA, 11), Guerrero (GRO, 12), Hidalgo (HID, 13), Jalisco (JAL, 14), State of Mexico (MEX, 15), Michoacán de Ocampo (MIC, 16), Morelos (MOR, 17), Nayarit (NAY, 18), Nuevo León (NLE, 19), Oaxaca (OAX, 20), Puebla (PUE, 21), Querétaro (QUE, 22), Quintana Roo (ROO, 23), San Luis Potosí (SLP, 24), Sinaloa (SIN, 25), Sonora (SON, 26), Tabasco (TAB, 27), Tamaulipas (TAM, 28), Tlaxcala (TLA, 29), Veracruz de Ignacio de la Llave (VER, 30), Yucatán (YUC, 31), Zacatecas (ZAC, 32)] for all the diseases under study. Significant differences between periods and federative entities were identified using Kruskal-Wallis and Mann-Whitney tests, with a significance level of p<0.05. For a preliminary view of the distribution of each disease by state, a map of the state political division at a scale of 1:250,000, available at [15], was used. Statistical analyses were performed using R v.3.6.2 (R Foundation for Statistical Computing, Vienna, Austria) [16], with additional libraries such as nparcomp v.3.0 [17], sf v.1.0-4 [18], ggplot2 v.3.3.2 [19], and patchwork v.1.1.2 [20].

Cluster analysis and data visualization

Cluster analysis was performed using heat map techniques and correlation analysis to visualize RD groupings and similarities between study periods. This two-dimensional tool, suitable for high-dimensional data, assigns numerical values to colors organized by similarity. An Unweighted Pair Group Method with Arithmetic Mean (UPGMA) dendrogram complemented the analysis, showing a cophenetic correlation of 0.91. These advanced procedures were performed using R, with specific libraries such as ggplot2, dendextend v.1.15.2 [21], and ComplexHeatmap v.1.10.2 [22].

## Results

Variation in the incidence of respiratory diseases

**Figure 1:**
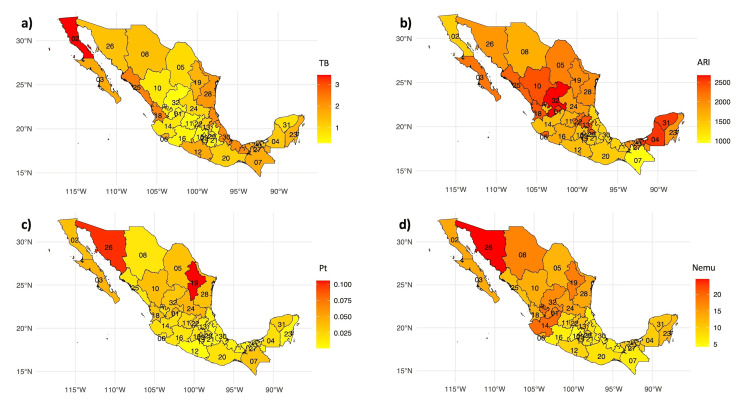
Average infection rate of respiratory diseases at the national level. a) Tuberculosis (TB), b) Acute respiratory infections (ARI), c) Pertussis (Pt), and d) Pneumonia-Bronchopneumonia (Nemu).

Our 21-year analysis of incidence trends for four key respiratory diseases in Mexico revealed statistically significant patterns (χ² = 63.57, p < 0.01), as shown in Figure 1.

Tuberculosis: preference for border and coastal states

A higher prevalence of tuberculosis was observed in border states, most notably Baja California Norte, and in coastal states with important seaports, including Veracruz, Tamaulipas, Tabasco, Guerrero, Sinaloa, and Nayarit. This distribution pattern suggests an influence of mobility and trade (Figure 1A). In contrast, the central states reported significantly lower infection rates.

Acute respiratory infections: high national incidence

ARIs displayed a more uniform distribution across the country, with high incidence rates in Aguascalientes, Zacatecas, Campeche, and Yucatán, indicating these as prevalence hotspots. Chiapas stood out for having the lowest ARI rate in the comparative analysis (Figure 1B).

Pertussis: specific incidence hotspots

Pertussis incidence was particularly high in Sonora and Nuevo León. Conversely, Guerrero, Puebla, and Quintana Roo exhibited lower rates, suggesting a heterogeneous distribution of this disease across the national territory (Figure 1C).

Pneumonia and bronchopneumonia: prevalence in the north

Northern border states, such as Sonora, Chihuahua, and Nuevo León, reported notably high incidences of pneumonia and bronchopneumonia. This trend emphasizes the need to focus health interventions in these regions. In contrast, the southern states showed lower rates (Figure 1D).

Seasonal variability in the incidence of respiratory diseases

The analysis of the mean monthly infection rates over two decades revealed significant differences and marked intramonthly variations among the four respiratory diseases studied (χ² = 89.57, p < 0.0001). These variations highlight the heterogeneity in the number of reported cases between states over the years, as well as a clear seasonal pattern in disease incidence.

Distinct seasonal patterns by disease

Seasonal analysis showed that the incidence and frequency of TB and Pt were highest in spring (27.4% and 71 cases per 100,000 population for TB; 32% and two cases for Pt) and summer (26.2% and 70 cases for TB; 29% and two cases for Pt), compared with fall (25.5% and 68 cases for TB; 23.7% and two cases for Pt) and winter (21.9% and 59 cases for TB; 16.1% and one case for Pt). However, there were no significant differences between seasons. In contrast, ARI showed frequencies of 106,612, 80,849, 118,894, and 131,840 cases in spring, summer, fall, and winter, respectively, corresponding to incidences of 24.5%, 18.5%, 27.3%, and 29.7%. Nemu showed frequencies of 634, 439, 671, and 909 cases with incidences of 24.4%, 16.9%, 25.8%, and 33% for the same seasons. Both ARI and Nemu had significantly higher incidences in winter (χ² = 9.23, p < 0.003), which coincides with the season with the highest prevalence of respiratory diseases overall.

Spatiotemporal correlation analysis using heat maps

**Figure 2:**
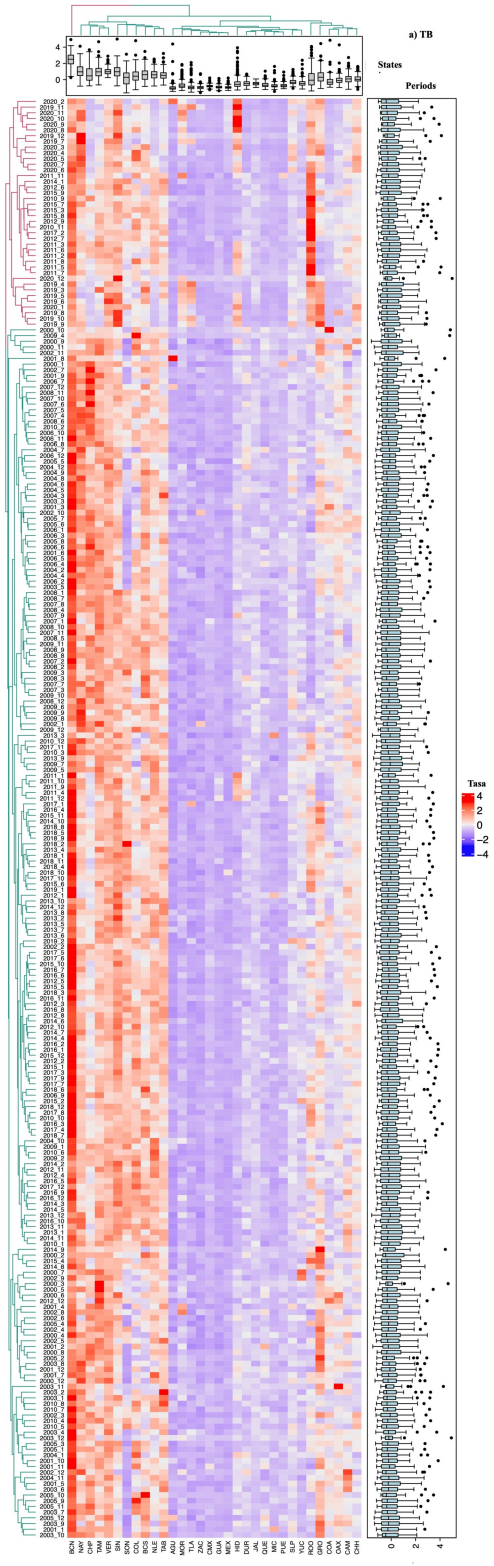
Heatmap based on the relative tuberculosis (TB) rate in each state of Mexico (in columns) for each analyzed period (in rows). The intense blue color represents the minimum value, increasing towards red. Aguascalientes (AGU, 1), Baja California (BCN, 2), Baja California Sur (BCS, 3), Campeche (CAM, 4), Coahuila de Zaragoza (COA, 5), Colima (COL, 6), Chiapas (CHP, 7), Chihuahua (CHH, 8), Mexico City (CMX, 9), Durango (DUR, 10), Guanajuato (GUA, 11), Guerrero (GRO, 12), Hidalgo (HID, 13), Jalisco (JAL, 14), State of Mexico (MEX, 15), Michoacán de Ocampo (MIC, 16), Morelos (MOR, 17), Nayarit (NAY, 18), Nuevo León (NLE, 19), Oaxaca (OAX, 20), Puebla (PUE, 21), Querétaro (QUE, 22), Quintana Roo (ROO, 23), San Luis Potosí (SLP, 24), Sinaloa (SIN, 25), Sonora (SON, 26), Tabasco (TAB, 27), Tamaulipas (TAM, 28), Tlaxcala (TLA, 29), Veracruz de Ignacio de la Llave (VER, 30), Yucatán (YUC, 31), Zacatecas (ZAC, 32)

**Figure 3:**
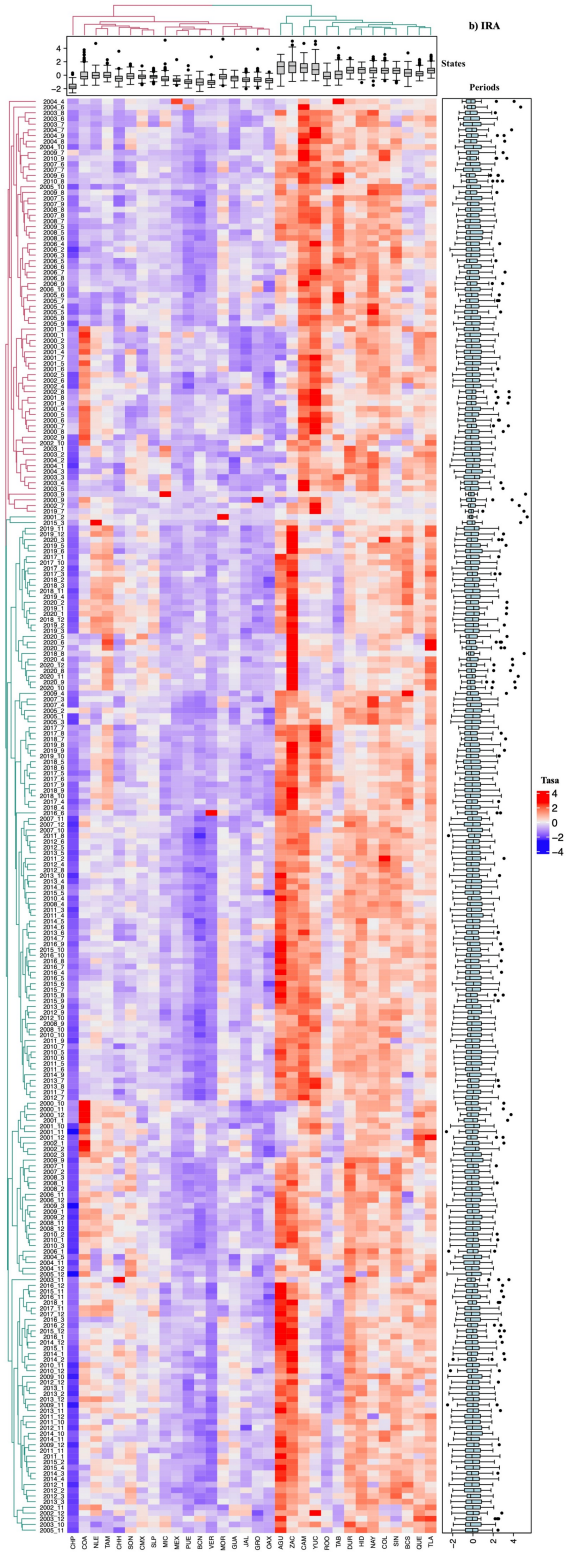
Heatmap based on the relative rate of Acute Respiratory Infections (ARIs) in each state of Mexico (in columns) for each analyzed period (in rows). The intense blue color represents the minimum value, increasing towards red. Aguascalientes (AGU, 1), Baja California (BCN, 2), Baja California Sur (BCS, 3), Campeche (CAM, 4), Coahuila de Zaragoza (COA, 5), Colima (COL, 6), Chiapas (CHP, 7), Chihuahua (CHH, 8), Mexico City (CMX, 9), Durango (DUR, 10), Guanajuato (GUA, 11), Guerrero (GRO, 12), Hidalgo (HID, 13), Jalisco (JAL, 14), State of Mexico (MEX, 15), Michoacán de Ocampo (MIC, 16), Morelos (MOR, 17), Nayarit (NAY, 18), Nuevo León (NLE, 19), Oaxaca (OAX, 20), Puebla (PUE, 21), Querétaro (QUE, 22), Quintana Roo (ROO, 23), San Luis Potosí (SLP, 24), Sinaloa (SIN, 25), Sonora (SON, 26), Tabasco (TAB, 27), Tamaulipas (TAM, 28), Tlaxcala (TLA, 29), Veracruz de Ignacio de la Llave (VER, 30), Yucatán (YUC, 31), Zacatecas (ZAC, 32)

**Figure 4:**
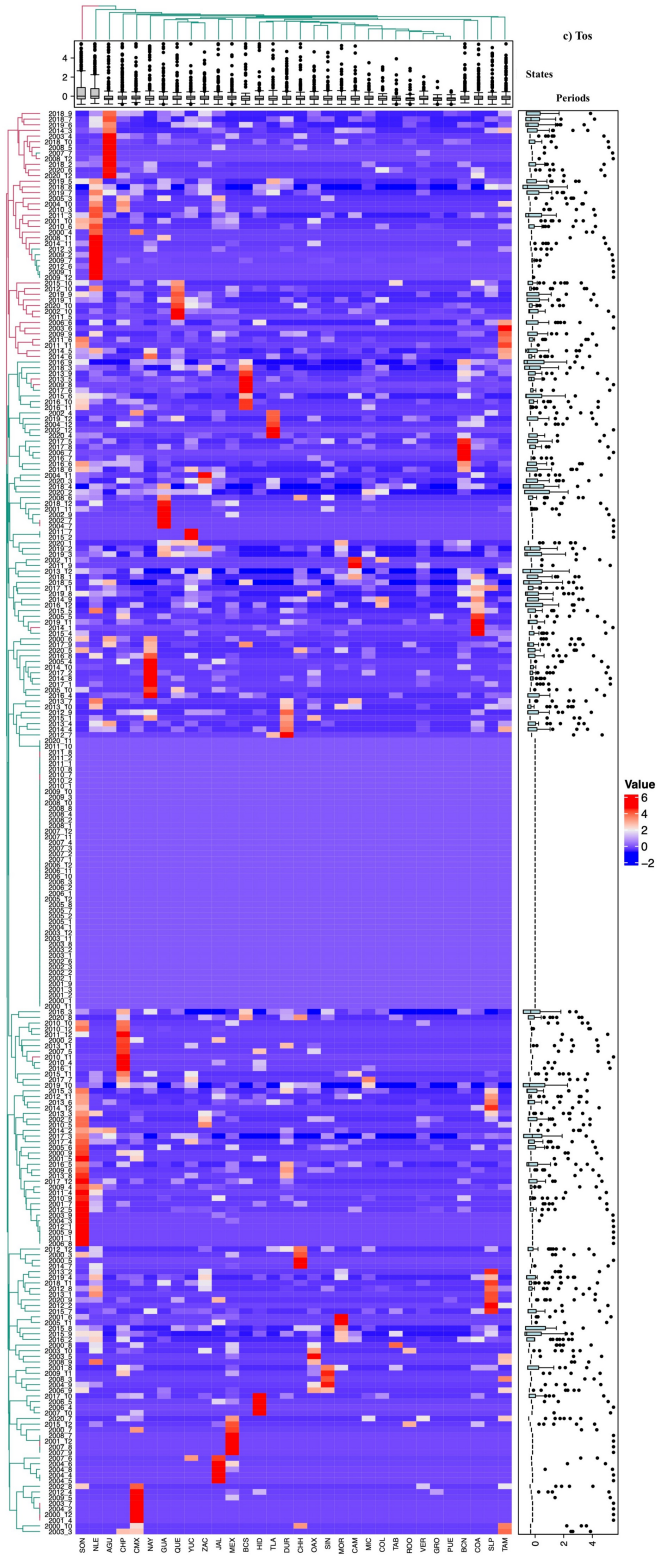
Heatmap based on the relative rate of Pertussis (Pt) in each state of Mexico (in columns) for each analyzed period (in rows). The intense blue color represents the minimum value, increasing towards red. Aguascalientes (AGU, 1), Baja California (BCN, 2), Baja California Sur (BCS, 3), Campeche (CAM, 4), Coahuila de Zaragoza (COA, 5), Colima (COL, 6), Chiapas (CHP, 7), Chihuahua (CHH, 8), Mexico City (CMX, 9), Durango (DUR, 10), Guanajuato (GUA, 11), Guerrero (GRO, 12), Hidalgo (HID, 13), Jalisco (JAL, 14), State of Mexico (MEX, 15), Michoacán de Ocampo (MIC, 16), Morelos (MOR, 17), Nayarit (NAY, 18), Nuevo León (NLE, 19), Oaxaca (OAX, 20), Puebla (PUE, 21), Querétaro (QUE, 22), Quintana Roo (ROO, 23), San Luis Potosí (SLP, 24), Sinaloa (SIN, 25), Sonora (SON, 26), Tabasco (TAB, 27), Tamaulipas (TAM, 28), Tlaxcala (TLA, 29), Veracruz de Ignacio de la Llave (VER, 30), Yucatán (YUC, 31), Zacatecas (ZAC, 32)

**Figure 5:**
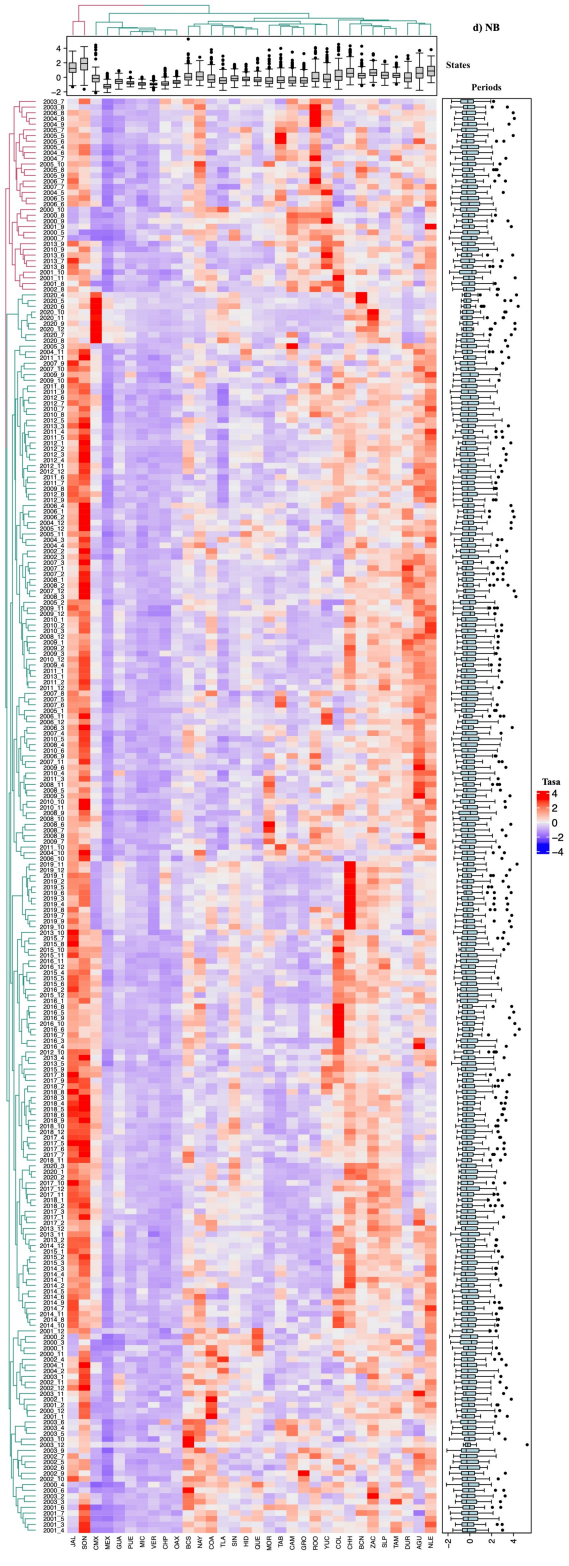
Heatmap based on the relative rate of Pneumonia-Bronchopneumonia (Nemu) in each state of Mexico (in columns) for each analyzed period (in rows). The intense blue color represents the minimum value, increasing towards red. Aguascalientes (AGU, 1), Baja California (BCN, 2), Baja California Sur (BCS, 3), Campeche (CAM, 4), Coahuila de Zaragoza (COA, 5), Colima (COL, 6), Chiapas (CHP, 7), Chihuahua (CHH, 8), Mexico City (CMX, 9), Durango (DUR, 10), Guanajuato (GUA, 11), Guerrero (GRO, 12), Hidalgo (HID, 13), Jalisco (JAL, 14), State of Mexico (MEX, 15), Michoacán de Ocampo (MIC, 16), Morelos (MOR, 17), Nayarit (NAY, 18), Nuevo León (NLE, 19), Oaxaca (OAX, 20), Puebla (PUE, 21), Querétaro (QUE, 22), Quintana Roo (ROO, 23), San Luis Potosí (SLP, 24), Sinaloa (SIN, 25), Sonora (SON, 26), Tabasco (TAB, 27), Tamaulipas (TAM, 28), Tlaxcala (TLA, 29), Veracruz de Ignacio de la Llave (VER, 30), Yucatán (YUC, 31), Zacatecas (ZAC, 32)

The interpretation of the heat maps generated for each respiratory disease revealed association patterns consistent with the results described previously. These maps visualize the spatial and temporal correlations between disease incidence in different states and periods through a color gradient, where intense blue indicates weaker correlations and red represents stronger correlations (Figures 2-5).

Groupings and subdivisions by state and period

Two main clusters were identified, differentiated by color (red and green), corresponding to varying levels of incidence and correlation between states and periods analyzed. Pt and Nemu exhibited more detailed subdivisions of these groups, particularly in comparisons between states, reflecting significant differences in incidence trends (Figures 4-5). Regarding study periods, clear group divisions were observed for all four diseases, with the formation of more than 100 subgroups for TB, Nemu, and ARIs, indicating significant temporal variations in incidence.

Incidence patterns and temporal variations

Additionally, states were organized in columns according to the relative incidence of cases, illustrating the distribution of mean relative rates through box plots. The arrangement of the columns, from left to right, shows states with high, medium, and low incidences. Similarly, study periods were categorized at the top based on their similarity in case incidence. For TB (Figure 2) and Pt (Figure 4), a notable increase in incidence was observed in the last decade, with significant peaks in 2019 and 2020 (χ² = 33.57, p < 0.02). In contrast, for ARI (Figure 3) and pneumonia (Figure 5), a reduction in the number of cases was documented, though this trend did not reach statistical significance (χ² = 23.1, p < 0.288).

Statistical significance and group differentiation

Chromatic differences within each group and between clusters indicated significant variations in disease incidence (p < 0.05), underscoring the usefulness of these analyses in identifying distribution patterns and relevant temporal changes.

## Discussion

Respiratory diseases are a major cause of mortality in Mexico, with approximately 80,000 deaths reported nationwide in 2015 [12]. Our study demonstrates an increase in RIs over the past decade, especially for TB and Pt, accompanied by a significant decrease in ARIs and Nemu. These findings align with previous reports by Soto-Estrada et al. [10], who also identified fluctuations in RI cases in Mexico from 1990 to 2014. However, the high subdivision of clusters during the study period and the disparities between states suggest a more complex behavior.

The increase in TB cases in 2019 and 2020 presents a national health challenge. This is particularly concerning because globally, the World Health Organization’s Regional Plan Strategy reported a downward trend in TB incidence [23]. In Mexico, this upward trend may be attributed to various factors, including social inequalities, socioeconomic variables, and differences in health service coverage among regions [24]. The containment measures implemented during the COVID-19 pandemic in 2019-2020 likely contributed to this increase due to the disruption of health services and preventive strategies. Close contact and overcrowding have been identified as key risk factors [25,26], which may have facilitated transmission during the pandemic.

Since 2006, Mexico has worked with the Pan American Health Organization (PAHO) to reduce TB prevalence and mortality as part of the Millennium Development Goals [23]. Although Mexico has historically controlled the disease, the recent increase in cases, especially in border states with high immigration, is concerning. The future management of TB will depend on control measures, population mobility, and public health policies. While TB detection, diagnosis, and treatment strategies have improved, coverage remains insufficient [27]. Strengthening early diagnosis, therapeutic control, and prevention, along with identifying individuals in close contact with TB patients, is essential to interrupt transmission [28].

Pt continues to be a significant health problem in Mexico [29,30]. Since 2009, there has been a notable increase in Pt cases, despite relatively low numbers in the previous decade. This trend mirrors earlier observations by Suárez-Idueta et al. [29], who documented an increase in 2009 in a study covering 1995-2011. Nuevo León and Sonora are particularly affected, with the State of Mexico also showing significant increases. It is reported that Pt does not follow a seasonal epidemiological pattern, with outbreaks every three to five years [29-32]. However, our study indicates a significant increase in disease prevalence over the last decade (2012-2019), suggesting that Pt is rising in Mexico, as seen in other regions of the Americas and Europe. The decline in Pt vaccination coverage, as reported by PAHO [33], alongside vaccine hesitancy and misinformation, may contribute to outbreaks [34].

Regarding the decrease in ARIs and Nemu over the last decade, while differences are observed between states - likely due to multifactorial causes [24] - the main distinction lies in the temporal scale and cluster subdivision during the study period. This aspect requires further investigation in future time series studies. The seasonal increase in cases during winter and fall aligns with previous research [11,35,36]. Unlike Escobar-Rojas et al. [11], who reported a decreasing trend between 1984 and 2010, our study indicates a reduction in these diseases over the last decade, suggesting that interventions to control these diseases are effective. However, recent temporal peaks (2018-2020) highlight the need to strengthen preventive measures, such as vaccination, and identify risk areas to support policy changes.

While this study provides valuable quantitative data on annual and state-level case numbers of different respiratory diseases in Mexico, it has limitations, such as the lack of additional variables that could enhance the understanding of disease behavior over time. Future studies should incorporate these variables to improve associations, correlations, and comparisons between states.

Overall, the study presents a concerning scenario of respiratory diseases in Mexico, with significant increases in TB, ARIs, and Pt over the past decade. These findings emphasize the importance of strengthening prevention strategies, early diagnosis, and control of respiratory diseases at the national level. Public health policies should focus on improving vaccination coverage, ensuring access to quality healthcare services across all regions, and raising public awareness about the importance of prevention and appropriate treatment. Collaboration among healthcare institutions, researchers, and the public is crucial to comprehensively and effectively address this issue.

## Conclusions

The study reveals a worrying upward trend in cases of respiratory diseases in Mexico over the past decade, with significant increases in TB and PT, especially in 2019 and 2020. These figures reflect challenges in prevention and control strategies for these diseases. In addition, regional disparities have been identified, with border states and those with important ports particularly affected. Pt shows a consistent increase, unrelated to seasonal patterns, associated with declining vaccination coverage and vaccine skepticism. The disruption of health services and preventive measures during the COVID-19 pandemic may have contributed to the increase in TB cases. However, some respiratory infections, such as ARIs and Nemu, have shown declining trends in recent years, indicating the effectiveness of specific preventive measures. This study highlights the need for more robust public health policies, including the promotion of vaccination, equitable access to health care, and public awareness regarding the prevention and treatment of respiratory diseases in Mexico.
